# Your Care Mitigates My Ego Depletion: Why and When Perfectionists Show Incivility Toward Coworkers

**DOI:** 10.3389/fpsyg.2021.746205

**Published:** 2021-11-11

**Authors:** Muhammad Ali Hussain, Lu Chen, Lusi Wu

**Affiliations:** ^1^School of Economics and Management, Centre of Western Africa Studies, University of Electronic Science and Technology of China, Chengdu, China; ^2^China Academy of Corporate Governance Nankai University, Tianjin, China

**Keywords:** self-oriented perfectionism, other-oriented perfectionism, incivility, ego depletion, empathic concern

## Abstract

Drawing on ego depletion theory and trait activation theory, this study examines why and when employee perfectionism personality is linked with incivility toward coworkers. The study indulges ego depletion as a mediator between perfectionism personality and incivility toward coworkers, with coworker empathic concern moderating the relationship between perfectionism personality and ego depletion. A three-waved questionnaire was incorporated with sample of 253 employee-coworker dyads. Our findings demonstrate that dimensions of perfectionism personality are positively associated with incivility toward coworkers. In addition, our study confirms that ego depletion mediates the relationship between self-oriented perfectionism, other-oriented perfectionism, and incivility toward coworkers. Furthermore, our study shows that high levels of coworker empathic concern weakens the direct effect of self-oriented perfectionism on ego depletion along with the indirect effect of self-oriented perfectionism on incivility toward coworkers. Theoretical and practical implications of the study are discussed in the organizational context.

## Introduction

*Workplace incivility* refers to low-intensity deviant behaviors with ambiguous intent to harm and violation of norms for mutual respect (Andersson and Pearson, 1999). It has received prominent attention among the researchers on its detrimental effects due to its prevalent nature in organizations and negative outcomes. For instance, workplace incivility has been documented to relates negatively to organizational citizenship behavior (Liu and Zhou, 2018), job satisfaction (Koon, 2017; Alola et al., 2018), intention to stay (Griffin, 2010), and employee performance (Chen et al., 2013). Additionally, studies have confirmed that incivility prompts behavioral strains that lead to adversarial effects like workplace deviance (Penney and Spector, 2005), intentional withdrawal (Sliter et al., 2012), turnover intentions (Huang and Lin, 2019), and increased employee cynical behaviors (Alola et al., 2019a). To better understand its antecedents and outcomes, it is necessary to differentiate individuals based on personality attributes that initiate patterns of uncivil conducts at the workplace and to explore potential ways to reduce its negative impacts.

Previous studies have explored employee incivility toward supervisors (Reio and Sanders-Reio, 2011), customers (Alola et al., 2019b), and coworkers (Walsh et al., 2018). Our research focuses on incivility toward coworkers because colleagues regularly interact with fellow employees in workplace settings and can have strong influences on relational associations (Umphress et al., 2003). Coworkers thus play a critical role as a social referent with whom the focal employee interacts (Takeuchi et al., 2011). The observation of coworkers that the focal employee engages in uncivil behaviors would shape their mental reactions, which in turn impacts employee-coworker exchange relationships. Following this research paradigm, previous empirical evidences have comprehended that leadership styles would predict the diminishing pattern of incivility from employee-coworker interaction perspective (Walsh et al., 2018). Given the prevalence of workplace incivility, a number of individual and contextual factors have been identified that ultimately invigorate uncivil behaviors in social affiliations (Meier and Semmer, 2013; Sears and Humiston, 2015; Lanzo et al., 2016). For instance, Lanzo et al. (2016) found that extensive working hours induce physical strain in individuals, leading them to engage in antisocial conducts. Further, research by Meier and Semmer (2013) argued that work characteristics such as lack of reciprocity result in negative emotional states, which exacerbate incivility. However, the role of the perfectionism personality of an employee in the process has been largely ignored. Thus, both researchers and practitioners need to identify the factors determining why incivility occurs in work settings.

Previous studies have found that personality of employees (Milam et al., 2009), emotional state (Reio and Ghosh, 2009), and aversive behaviors (Lata and Chaudhary, 2020) are strong antecedents of incivility. They reported that individuals conduct incivility when prioritizing ideal image for oneself (narcissist) and unveiling moral disengagement (machiavellianism), which in turn influences the interpersonal connections (Turnipseed and Landay, 2018). Traditionally, the empirical evidence has demonstrated that individuals respond differently toward negative workplace practices like incivility by accounting their personal experiences (Beattie and Griffin, 2014). However, empirical evidence on the relationship between the personality traits of an individual and workplace incivility are rather limited. The first purpose of the present study is thus to investigate whether perfectionism personality, as a multidimensional construct, can shape the incivility of an individual in the workplace.

*Perfectionism personality* refers to one’s own identity that detains incompatible targets, demanding flawless performance and rigorous self-criticism (Frost et al., 1990; Dunkley et al., 2003; Flaxman et al., 2012). Prior research has shown that the perfectionist manifests negative demeanors by adhering to display perfection, which affects relational associations (Vicent et al., 2017). Further, they attenuate interpersonal connections, reporting antisocial behaviors, and escalate dysconnectivity in social settings (Hewitt et al., 2006). For instance, Dang et al. (2020) have found that employees with high perfectionism greatly emphasize disagreement between their demanded flawless performance and their genuine performance, and tend to convey disruptive behaviors in interpersonal communications. Reis and Prestele (2020) also suggested that perfectionism personality increases psychological costs by engaging individuals in abusive behaviors at workplace. Nevertheless, very few studies have explored whether, why, and when perfectionism personality of employees lead to incivility toward their coworkers.

Ego depletion theory advocates that behaviors of employees are mainly formed by the decrement of self-regulatory resources and restraint over impulses, emotions, or desires of an individual (Baumeister et al., 1998). The depletion of self-regulatory resources attenuates the strength of employees to deal with a set of different exercises that exceptionally demand self-control in relational settings (Qian et al., 2020), which further causes uncivil or adverse results. Because perfectionism personality is connected with negative emotional states (i.e., depression, stress, and anxiety, Flett et al., 1991; Ulu and Tezer, 2010), perfectionists are more likely to experience less control and are turning to be ego depleted. Furthermore, ego-depleted perfectionists are more psychologically detached and feel incompetent to oversee negative impulses or antagonistic motivations in their relational association, which leads to uncivil behaviors in the workplace (Zhang et al., 2019; Qian et al., 2020). Therefore, the second objective of our study is to explore how different conceptions of perfectionism drive individuals to incivility in interpersonal interactions from a viewpoint of ego depletion. Specifically, we propose that ego depletion plays a mediating role between the perfectionism personality and incivility of employees toward their coworkers.

Moreover, the trait activation theory suggests that perfectionism personality directs the outflow of the emotions and behaviors of employees, but circumstances also impact and shape emotions and behaviors by sending pertinent or prohibitive signals (Tett and Burnett, 2003). Empathic concern refers to “the other-oriented emotion elicited by and congruent with the perceived welfare of someone else in need” (Batson, 2011, p.11), and is highly effective in unfavorable circumstances and negative emotional states (Batanova and Loukas, 2011). Previous studies have shown that empathic concern from other people can alleviate negative emotions and mental states of individuals (Bussey et al., 2015; Wu et al., 2020). Concern and care from coworkers can safeguard the affected perfectionists to be less psychologically depleted, and thus helps them reduce negative perceptions and hostility toward their coworkers during interactions. Therefore, the third goal of our study is to examine whether coworker empathic concern can attenuate the mediating effects of ego depletion on the relationship between perfectionism personality of employees and their incivility toward their coworkers.

To summarize, the present research contributes to the extant literature in various ways. First, we explore the dark side of perfectionism personality from an employee-coworker interaction viewpoint by examining its negative consequences on workplace incivility. Second, on the basis of ego depletion theory (Baumeister et al., 1998), we investigate the mediating roles of negative emotional state (i.e., ego depletion) between employee perfectionism personality and incivility toward coworkers. Third, from trait action perspective (Tett and Burnett, 2003), we introduce coworker empathic concern as a contingency and examine its moderating effects on the mediation mechanism of ego depletion in between perfectionism personality and incivility. Collectivistic Pakistani culture, as recommended by Hofstede (1980), has innated abilities and societal norms to work for the well-being of others by displaying high empathic concern. Additionally, it also buffers the negative influence and its relevant consequences during interpersonal interactions. The current study also draws significant attention among the organization practitioners to consider personality characteristics of individuals as being highly important in reshaping their mindset to unveil incivility toward a coworker.

## Theory and Hypotheses

### Perfectionism Personality and Incivility Toward Coworkers

*Perfectionism* is a personality trait of setting perfect standards in work, often accompanied with excess criticism in self-estimate (Hewitt and Flett, 1991). Earlier studies have focused on the unidimensional aspect of perfectionism, which claims that perfectionists are more prone to suffer from psychiatric problems when they are under stress (Hewitt and Dyck, 1986; Flett et al., 1989). Increasing evidence has highlighted the fact that perfectionism is primarily conceptualized as a multidimensional construct, particularly as different conceptions of perfectionism. It might have varied and sometimes had contradictory associations with the indicators of social isolation and emotion regulation (Stoeber et al., 2017). In accordance, we specifically follow the multidimensional perfectionism model of Hewitt and Flett (1991), which comprises three dimensions of perfectionism: self-oriented perfectionism, other-oriented perfectionism, and socially prescribed perfectionism (Hewitt and Flett, 1991). Specifically, *self-oriented perfectionism* is the setting of highly irrelevant goals for oneself, *other-oriented perfectionism* expresses unrealistic expectations and standards for others, and *socially prescribed perfectionism* is when an individual holds the belief that others anticipate perfection from him or her (Hewitt and Flett, 1991). We propose that each dimension of perfectionism personality will have a negative impact on incivility toward coworkers.

First, employees high in self-oriented perfectionism are characterized by the critical self-expectation of an individual to execute perfection in their work. They would focus on their personal achievements without caring about organizational standards, which may stimulate negative effects. In addition, evaluation of their own performance may induce the feelings of rigorous self-criticism, and self-discipline. Self-oriented perfectionism has been primarily considered as a dysfunctional personality trait (Hewitt and Flett, 1991), mainly linked with various adverse outcomes such as depression (Flett et al., 1991; Smith et al., 2016), hostility, and anger (Besser et al., 2004; Blankstein and Lumley, 2008; Stoeber et al., 2014). According to ego depletion theory, employees with high self-oriented perfectionism would feel stressed and experience resource loss due to their irrational objectives. Consequently, they would feel suppressed and disappointed when they have drained resources, which would lead to incivility toward coworkers.

Second, other-oriented perfectionism is a personality trait that expresses unrealistic expectations and standards for others (Hewitt and Flett, 1991). Individuals with other-oriented perfectionism are profoundly focused on negative behaviors of others rather than their own. When employees of high other-oriented perfectionism set a critical evaluation for others to meet their standards, they may act impolitely due to interpersonal conflicts (Stoeber, 2014), and become more engaged in antisocial behaviors (Stoeber et al., 2017), thereby showing less compassion toward others (Stoeber et al., 2020). Therefore, employees who exhibit other-oriented perfectionism are highly focused on requirements of other people, restraining them from following their instincts, depleting self-capabilities of employees, and nurturing negative emotions. Following the theoretical perspective of ego depletion, it traverses that other-oriented individuals are more prone to exhibit ruthless behaviors such as incivility toward coworkers.

Finally, socially prescribed perfectionism depicts individuals who believe others expect perfection of them and that others will be highly critical of them if they are unproductive (Hewitt and Flett, 1991). Employees who expound socially prescribed perfectionism hold the conviction that others have set incompatible goals for one to exercise and that others will emphasize immaculate execution, which cause them to perform ineffectively (Ferrari and Mautz, 1997). Inevitably, socially prescribed perfectionists feel powerless and desperate, as it is arduous to live up to the high expectations of others (Stoeber, 2014). In adhering to reveal perfection, the rational thinking for such employees reduces due to limited decision making power, which has a propensity to be psychologically detached and more vulnerable to depressive symptoms (Newman et al., 2019; Smith et al., 2020). In response, socially prescribed perfectionism employees feel anxious to meet expectancies of society to be perfectionists. In the vein of ego depletion theory, the socially prescribed trait will invoke the negative sentiments in the form of fear and loss of inner willpower, with extraneous factors to be greatly imposed by others. Thus, they feel distressed and socially isolated when faced with destructive criticism, which leads them to engage in negative behaviors such as incivility toward coworkers. Indeed, prior studies have demonstrated that socially prescribed perfectionists display more antisocial behaviors and hostility (Stoeber et al., 2017). Hence, we hypothesize that,

H1: (H1a) Self-oriented perfectionism, (H1b) other-oriented perfectionism, and (H1c) socially prescribed perfectionism has a positive influence on incivility toward coworkers.

### The Mediating Role of Ego Depletion

Ego depletion is explained as “a temporary reduction in the capacity of the self or willingness to engage in volitional action caused by the prior exercise of volitional” (Baumeister et al., 1998, p. 1253). According to ego depletion theory, perfectionist individuals would experience the phase of depletion due to psychological factors such as experiencing distress, evolving repressing thoughts, and additional obligations impairing the self-control of the individual induced by his or her personality. Besides, depleted individuals may undermine positive thoughts, which could result in negative behaviors such as incivility toward colleagues. We expect that each dimension of perfectionism personality will prompt incivility toward coworkers through ego depletion.

Specifically, the self-oriented perfectionism employees will exercise a range of narrow-minded behaviors such as self-promotion over peers, consistency in their performances, and setting exacting proportions of achievement for themselves. To meet up their expectations, employees encounter a shortage of resources and reduce their willpower to voluntarily practice their workplace (Baumeister et al., 1998, 2007; Muraven and Baumeister, 2000). Thus, self-oriented perfectionism employees lose their capacity to regulate the resources required and experience negative sentiments such as ego depletion. Likewise, the other-oriented perfectionism employees exceptionally centered around the execution of others. These employees pinpoint the weak point of others rather than discuss the deficiencies of self, which ultimately impacted the interpersonal relationships at workplace (Flett et al., 2001–2002). Such employees will fundamentally assess as per perfectionistic concerns determined by others and they envision that others will submit to these norms accordingly. Consequently, the other-oriented perfectionism employees feel depleted, and this depletion immunizes through internal sources due to undeniable compliance and fulfillment of tasks imposed by others. Finally, the socially prescribed perfectionism employees encompass outwardly driven standards that inhibit the mental energy of individuals to be perfect and attempting to keep up ideal work attributes. Such employees hold that others will exceptionally critical of them if their performances are not up to the mark (Flett et al., 1991). Therefore, the socially prescribed perfectionism employees will experience ego depletion due to situational factors such as high work demands and excessive workload, which reduces their ability to exercise will power.

When employees face the resource loss and experience ego depletion due to limited self-control, they are prone to act unethically to perform subsequent tasks (Mead et al., 2009; Gino et al., 2010; Kouchaki and Smith, 2014). In our case, the perfectionists encounter less control at workplace, and they have to face up with ego depletion (Guo et al., 2020), which then leads to uncivil behaviors toward coworkers.

Specifically, the self-oriented perfectionism is indirectly associated with incivility toward coworkers *via* ego depletion because self-oriented personality attributes restrain individual mental capabilities to outperform, which triggers the individuals to display negative impulses such as incivility toward coworkers. Likewise, the other-oriented individuals demonstrate the perfectionistic standards fixed by other, not by oneself (Stoeber, 2014; Smith et al., 2018), which reduces resourcefulness and depletes the strength of an individual, which in turn increases incivility toward coworkers. Lastly, the socially prescribed perfectionism employees feel stressed and restless to be “perfect” by keeping the other norms in their intellect. Therefore, employees will encounter coercion depletion stage to regulate the resources and accelerate negative emotions in interpersonal connections such as incivility toward coworkers. Taken together, we hypothesize that,

H2: Ego depletion mediates the relationship between (H2a) self-oriented perfectionism, (H2b) other-oriented perfectionism, (H2c) socially prescribed perfectionism, and incivility toward coworkers.

### The Moderating Role of Empathic Concern

*Empathy* is characterized as an ethical feeling urged from internal emotions to help individuals in difficult situations, discussing distress for others as encountering the similar phase (Batson et al., 1988). *Empathic concern* refers to affective empathy that encompasses sympathetic attitude toward others and oversight of the individuals concerning their point of view (Davis, 1980, 1983; Chowdhury and Fernando, 2014). Essentially, the few previous studies enlightened the conception of empathic concern as a highly important personality trait, to subsequently lessen aggression and diminish counterproductive behaviors (Batanova and Loukas, 2011; Ho and Gupta, 2012). Based upon trait activation theory (Tett and Guterman, 2000; Tett and Burnett, 2003), we suggest that empathic concern shown by coworkers is an integral situational constituent that can moderate the impacts of different conceptions of perfectionism personality on employee ego depletion. In particular, we expect that empathic concern of coworkers would dampen the positive association between each dimension of perfectionism personality on ego depletion.

Ho and Gupta (2012) contended that colleagues who have higher empathic concern tend to express high levels of care in interpersonal relations to overcome negative emotions. Individuals high in empathic concern usually have compassionate feelings and exhibit positive expressions in ethical situations (Pohling et al., 2016) and in attempting to uncover pessimistic emotional states, thereby, evading negative outcomes (Bussey et al., 2015; Wu et al., 2020). Accordingly, the empathic concern is highly helpful to overcome disruptive behaviors by fortifying the will power of individuals (Fang et al., 2020). Similarly, empathic concern of coworkers can bring psychological resources to perfectionists and reduce their depletion, and, consequently, employees would be less inclined to display uncivil behaviors.

In particular, coworkers with high empathic concern are considerate to reduce the selfish and self-centered concerns evicted by employees of self-oriented perfectionism and its consequences (Hewitt and Flett, 1991). We suggest that employees who perceive a kinder and optimistic attitude from their coworkers would show a weakened negative influence of self-oriented perfectionism on ego depletion. Similarly, the coworkers with high empathic concern develop strong interpersonal connections to mitigate destructive criticism under other-oriented perfectionism and its related emotional reactions (Stoeber, 2014). Consequently, we emphasize that for employees who receive essential care from their respective colleagues, the relationship between other-oriented perfectionism and ego depletion should be weaker. Additionally, employees with colleagues who protect the employees from resource loss situations would avoid experiencing ego depletion. Likewise, the regard of coworkers for their fellow employees by giving enough mental resources to exercise self-control in order to be less depleted. Therefore, we argue that when employees get empathy from their coworkers, the negative impact stimulated by socially prescribed perfectionism on ego depletion ought to be weaker. In short, high empathic individuals easily fit themselves in the shoes of another and thereby inhibit negative impulses like depletion.

Contrary to this, individuals with low empathic concern are genuinely less courteous and relatively less helpful in organizational settings (Kalshoven et al., 2013). Particularly, lower level of coworker empathic concern will strengthen the association between self-oriented perfectionism and ego depletion since coworkers cautiously notice the personal interests of their fellow employees. In fact, colleagues might consider the actions of those employees as granted rather than articulating kindness toward them due to their self-centered nature, thus heightening employee state of ego depletion. Subsequently, the lower levels of empathic concern will reinforce the negative influence of other-oriented perfectionism on ego depletion, due to lack of collaboration and active participation form their colleagues. For coworkers with low empathic concern are likely to prioritize their own preferences rather than displaying positive inclinations toward them, the unfavorable impact of socially prescribed perfectionism on ego depletion would be enhanced. Likewise, the low coworker empathic concern is considered to enlarge societal expectations under socially prescribed perfectionism which induces negative emotions like ego depletion. Therefore, the colleagues tend to show less care and sympathetic attitude toward those employees who are high in socially prescribed perfectionism, and, as a result, they lead to higher self-depletion. Accordingly, we hypothesize that,

H3: Coworker empathic concern moderates the positive relationship between (H3a) self-oriented perfectionism, (H3b) other-oriented perfectionism, (H3c) socially prescribed perfectionism, and ego depletion such that the relationship is weaker when coworker empathic concern is higher rather than lower.

The arguments mentioned earlier provide support for a comprehensive framework in which ego depletion mediates the relationships between dimensions of perfectionism personality and incivility toward coworkers, and coworker empathic concern moderates the association between perfectionism personality conceptions and ego depletion. As per our proposition, coworker empathic concern serves as a moderator between perfectionism personality and ego depletion. Considering that ego depletion predicts incivility toward coworkers, it is insightful to draw that coworker empathic concern attenuates the positive indirect effect of ego depletion in order to measure the association between perfectionism personality and incivility toward coworkers, incorporating the moderated mediation model (Muller et al., 2005). As already narrated above, relationships between dimensions of perfectionism personality and ego depletion will be weaker when encountering high coworker empathic concern and indirect linkage of perfectionism personality on incivility toward coworkers *via* ego depletion would also be weakened among such individuals. Notably, when coworkers with high empathic concern express positive emotions such as feelings of kind heartedness toward perfectionist individuals to be less depleted, they eventually undermine the negative behaviors, such as incivility toward coworkers. On the other hand, when coworkers expressing low empathic concern are generally less conscious and showing less care toward depleted individuals, the indirect effect of perfectionism personality on incivility toward coworkers should be stronger. Accordingly, we hypothesize that:

H4: Coworker empathic concern moderates the indirect effect between (H4a) self-oriented perfectionism, (H4b) other-oriented perfectionism, (H4c) socially prescribed perfectionism, and incivility toward coworker *via* ego depletion such that the relationship is weaker among individuals who have higher empathic concern rather than lower.

**FIGURE 1:**
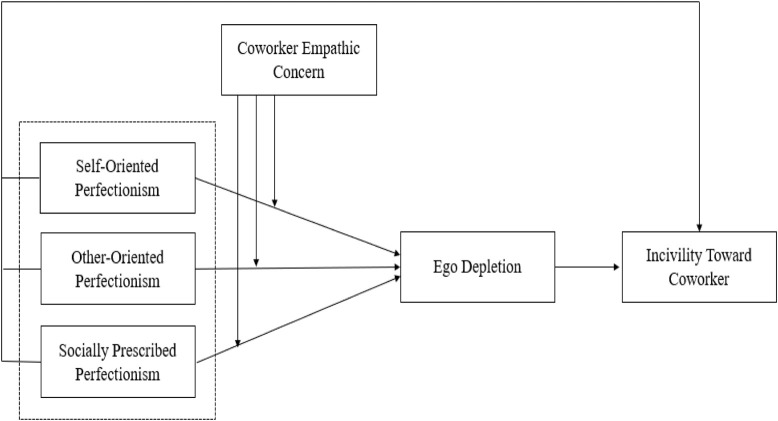
Theoretical model.

The overall theoretical model is shown in Figure 1.

## Materials and Methods

### Sample and Procedures

Data were collected from employees of a telecommunication company in Pakistan. Initially, the human resources department was requested for employee roster to assign a unique code for each employee. HR department then helped us to distribute the surveys among employees and their *coworkers*. Coworkers were employees who work in the same unit or division as the focal participants. A cover letter was appended with each questionnaire to illustrate the purpose of the study and encourage voluntary participation. The survey responses were enveloped and returned directly to the first author, to ensure the confidentiality of the survey results.

The survey was administered in three waves, with a 3-week time interval. At Time 1 (T1), we distributed questionnaires to 416 employees and 416 coworkers, out of which 318 employee-coworker matched responses were collected, with a response rate of 76.44%. In T1 survey, employees provided information on their perfectionism and demographics, and coworkers rated their empathic concern. At Time 2 (T2), we dispensed the surveys to all 318 employees who have completed T1 survey and received 284 responses, with a response rate of 89.30%. Participants evaluated their ego depletion in this survey. At Time 3 (T3), we disseminated the survey to coworkers of the 284 participants who completed T2 surveys. They rated their perception of incivility from the focal employees. After discarding the invalid and incomplete responses, we got 253 matched responses for employees and coworkers with an effective response rate of 60.82%.

In the final sample, 72% of employees were male, the average age was 37.64 years (SD = 6.67). A percentage of 57.3% of the employees have a bachelor’s degree, while 32.8% have a master’s degree. With respect to coworkers, 83.4% were male with the average age at 39.44 years (SD = 5.49). The majority of the coworkers have a master’s degree while about 51.8 and 36.8% had a bachelor’s degree.

### Measures

For all the investigated measures in this study, we encompassed a six-point Likert scale ranging from: 1 = (strongly disagree) and to 6 = (strongly agree).

#### Perfectionism

We measured perfectionism using a 15-item scale developed by Hewitt and Flett (1991). It is comprised of three dimensions. Self-oriented perfectionism was measured using five items. One sample item was “It makes me uneasy to see an error in my work” (α = 0.87). Other-oriented perfectionism was evaluated using five items. One sample item was “I have high expectations for the people who are important to me” (α = 0.86). Socially prescribed perfectionism was measured by five items. One sample item was “The better I do, the better I am expected to do” (α = 0.91).

#### Ego Depletion

To measure ego depletion, we indulged an 11-item scale developed by Salmon et al. (2014). One sample item was, “After I have worked very hard at something, I am not good at reloading to start a new task” (α = 0.95).

#### Empathic Concern

Empathic concern was assessed using a seven-item scale adapted from Davis (1983). A sample item was, “When I see my coworkers being taken advantage of, I feel kind of protective toward them” (α = 0.95).

#### Incivility Toward Coworkers

We incorporated a seven-item scale adapted from Cortina et al. (2001) to measure incivility toward coworker. One sample item was “My coworker put me down or are condescending to me” (α = 0.94).

#### Control Variables

Following prior research (Penney and Spector, 2005; Cortina et al., 2013), we included demographic characteristics such as gender, number of children, and number of hours worked per week as control variables in this study. Gender was treated as dummy variables and had two categories: 1 = (male) and 2 = (female) and the number of children was measured as a continuous variable. The number of hours worked per week was treated as a quantitative variable.

#### Analytical Strategy

We first ran a confirmatory factor analysis (CFA) to assess the measurement model. Path analyses was conducted to test our hypotheses. The indirect effects were tested using 10,000 bootstrapped samples as recommended by Preacher et al. (2007). All analyses were conducted using Mplus 7.4 (Muthén and Muthén, 2012).

Concerning the first level moderation, we performed the simple slope analysis to estimate the simple slopes at high (1 SD above the mean) and low (1 SD below the mean) by drawing 10,000 bootstrapped samples to test the hypothesized relationships. Particularly, to investigate the hypothesis H3 (a), *ego depletion* was regressed on all the controlled variables, independent variable (i.e., *self-oriented perfectionism*) and moderator (i.e., *coworker empathic concern);* for H3 (b), ego depletion was regressed on all the controlled variables, independent variable (i.e., other-oriented perfectionism) and moderator (coworker empathic concern); and for H3 (c), ego depletion was regressed on all the controlled variables, independent variable (socially prescribed perfectionism), moderator (coworker empathic concern), and their possible interactions accordingly. Before creating the interaction terms the predictors and moderating variables were mean-centered.

## Results

### Preliminary Analyses

We performed a series of confirmatory factor analysis (CFA) using Mplus 7.4 (Muthén and Muthén, 2012) to inspect the distinctiveness of all the incorporated study variables for self-oriented perfectionism, other-oriented perfectionism, socially prescribed perfectionism, coworker empathic concern, ego depletion, and incivility toward coworker. As presented in Table 1, our six-factor model showed best fit: χ^2^(725) = 1111.89, Comparative Fit Index (CFI) = 0.94, Tucker-Lewis Index (TLI) = 0.94, Root Mean Square Error of Approximation (RMSEA) = 0.04, and Standardized Root Mean Square Residual (SRMR) = 0.04, which was significantly better than the other nested models incorporating five-factor model [Δχ^2^ (Δdf) = 1127.74(5), *p* < 0.001], a four-factor model [Δχ^2^ (Δdf) = 758.74(4), *p* < 0.001], a three-factor model [Δχ^2^ (Δdf) = 419.57(3), *p* < 0.001], a two-factor model [Δχ^2^ (Δdf) = 459.83(2), *p* < 0.001], and subsequently one-factor model [Δχ^2^ (Δdf) = 658.65(1), *p* < 0.001]. The comprehensive details of fit indices were presented in Table 1. These above results provide enough support for the discriminability of the variables.

**TABLE 1:** Comparison of structural models.

Models	Factors	χ*^2^*	*d.f.*	χ*^2^/d.f.*	*CFI*	*TLI*	*RMSEA*	*SRMR*
Model 1	*Six factors:* Self-Oriented Perfectionism, Other’s Oriented Perfectionism, Socially Prescribed Perfectionism, Ego depletion, Coworker Empathic Concern and Incivility toward coworkers	1,111.89	725	1.53	0.94	0.94	0.04	0.04
Model 2	*Five factors:* Ego depletion and Incivility toward coworker combined into one factor	2,239.63	730	3.06	0.78	0.77	0.08	0.09
Model 3	*Four factors*: Self-Oriented Perfectionism, Other’s Oriented Perfectionism and Socially Prescribed Perfectionism combined into one factor	2,998.37	734	4.08	0.77	0.76	0.08	0.09
Model 4	*Three factors*: Self-Oriented Perfectionism, Other’s Oriented Perfectionism and Socially Prescribed Perfectionism combined into one factor; Ego depletion and Incivility toward coworker combined into one factor	3,417.94	737	4.63	0.61	0.59	0.11	0.12
Model 5	*Two factors:* Self-Oriented Perfectionism, Other’s Oriented Perfectionism and Socially Prescribed Perfectionism combined into one factor; Ego depletion, Coworker Empathic concern and Incivility toward coworker combined into one factor	3,877.77	739	5.24	0.55	0.52	0.12	0.13
Model 6	*One factor*: All variables combined into one factor	4,536.42	740	6.13	0.46	0.43	0.13	0.15

*χ^2^, chi-squared value; d.f., degrees of freedom; CFI, Comparative Fit Index; TLI, Tucker-Lewis Index; RMSEA, Root mean square error of approximation; SRMR, standardized root mean square residual.*

### Hypotheses Testing

Table 2 presents the means, standard deviations, bivariate correlations, and Cronbach alphas among the study variables.

**TABLE 2:** Means, standard deviations (SD) and correlations.

Variables	M	SD	1	2	3	4	5	6	7	8	9
1.Sudordinate Gender	1.28	0.44									
2.Number of Children	2.39	1.36	−0.15*								
3.Working Hours	45.39	2.86	−0.13*	−0.05							
4.Self-oriented perfectionism (T1)	3.86	1.41	0.11	−0.09	0.05	(0.87)					
5.Other-oriented perfectionism (T1)	3.58	1.28	−0.00	−0.00	0.07	0.22**	(0.86)				
6.Socially prescribed perfectionism (T1)	3.82	1.47	0.07	−0.05	0.13*	0.29**	0.23**	(0.91)			
7.Cowoker empathic concern (T1)	3.84	1.08	−0.00	0.00	−0.08	−0.10	0.01	−0.09	(0.95)		
8.Ego depletion (T2)	3.78	1.37	0.04	−0.10	0.11	0.28**	0.13*	0.56**	−0.10	(0.95)	
9.Incivility toward coworker (T3)	3.67	1.45	−0.00	−0.05	0.08	0.31**	0.12*	0.31**	−0.13*	0.45**	(0.94)

N = 253; Internal reliabilities (Cronbach alpha coefficients are specified along the diagonal parenthesis), **correlation is significant at the 0.01 level (two-tailed), and *correlation is significant at the 0.05 level (two-tailed).

Table 3 shows the unstandardized path coefficients for direct and indirect effects. Self-oriented perfectionism had a significant positive influence on incivility toward coworkers (*b* = 0.31, SE = 0.06, *p* < 0.001). Moreover, the other-oriented perfectionism had a significant positive impact on incivility toward coworkers (*b* = 0.17, SE = 0.05, *p* < 0.01). Subsequently, the socially prescribed perfectionism had a significant positive effect on incivility toward coworkers (*b* = 0.13, SE = 0.05, *p* < 0.05). Hence, the hypotheses H1 (a), H1 (b), and H1 (c) were supported. As presented in Table 3, ego depletion significantly mediated the relationship between self-oriented perfectionism and incivility toward coworkers [indirect effect = 0.19, 95%CI (0.12, 0.28)], strongly supporting H2 (a). Furthermore, the other-oriented perfectionism had a significant indirect effect on incivility toward coworkers *via* ego depletion [indirect effect = 0.10, 95%CI (0.03, 0.17)], supporting H2 (b). Opposing to our expectation, the association between socially prescribed perfectionism and incivility toward coworkers was not significantly mediated through ego depletion [indirect effect = 0.05, 95%CI (−0.03, 0.13)]. Thus, hypothesis H2 (c) was not supported.

**TABLE 3:** Bootstrapping results for unstandardized indirect effects from SEM.

Path	Coeff.	SE	95% CI	Path	Indirect effect	95% CI
SOP→WIC	0.31***	0.06	(0.19, 0.43)	SOP→ED→WIC	0.19***	(0.12, 0.28)
OOP→WIC	0.17**	0.05	(0.06, 0.28)	OOP→ED→WIC	0.10**	(0.03, 0.17)
SPP→WIC	0.13*	0.05	(0.02, 0.24)	SPP→ED→WIC	0.05	(−0.03, 0.13)

N = 253; ***p < 0.001, **p < 0.01, *p < 0.05; SOP, self-oriented perfectionism; OOP, other-oriented perfectionism; SPP, socially prescribed perfectionism; WIC, incivility toward coworker; ED, ego depletion; CI (95% Confidence Interval for Bootstrapping with 10,000 subsamples).

**FIGURE 2:**
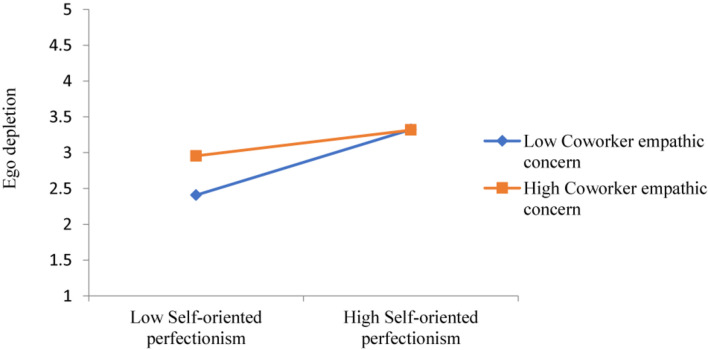
Interactive effect of self-oriented perfectionism and coworker empathic concern on ego depletion.

Hypothesis H3 (a) posits that the association between self-oriented perfectionism and ego depletion is moderated by coworker empathic concern. As reported, Table 4 delineates the significant interactive effect between self-oriented perfectionism and coworker empathic concern on ego depletion (*b* = −0.14, SE = 0.04, *p* < 0.01). Figure 2, illustrates that, the simple slope of self-oriented perfectionism on ego depletion was steeper when coworker empathic concern was low (*b* = 0.47, SE = 0.17, *p* < 0.01) rather than high (*b* = 0.17, SE = 0.18, ns), supporting H3 (a). However, the coworker empathic concern did not significantly moderate the relationship of other-oriented perfectionism (*b* = −0.02, SE = 0.04, ns) and socially prescribed perfectionism (*b* = −0.03, SE = 0.05, ns) with coworker empathic concern on ego depletion. Thus, hypotheses H3 (b) and H3 (c) were not supported.

**TABLE 4:** Regression results for interaction effects.

	Model 1			Model 2			Model 3		
Variables	Coeff.	SE	95%CI	Coeff.	SE	95%CI	Coeff.	SE	95%CI
**Mediator variable model with ego depletion as dependent variable**									
*Control variables*									
Subordinate gender	−0.06	0.07	(−0.18, 0.09)	−0.05	0.08	(−0.18, 0.10)	−0.07	0.08	(−0.21, 0.07)
Number of children	−0.13	0.11	(−0.36, 0.08)	−0.04	0.13	(−0.28, 0.22)	−0.05	0.13	(−0.29, 0.20)
Working hours	−0.14**	0.05	(−0.24, −0.04)	−0.09	0.06	(−0.20, 0.02)	−0.10	0.06	(−0.022, 0.00)
*Independent variables*									
Self-oriented perfectionism (SOP)	0.32***	0.06	(0.20, 0.43)						
Other-oriented perfectionism (OOP)				0.14**	0.06	(0.03, 0.25)			
Socially prescribed perfectionism (SPP)							0.54	0.06	(−0.07, 0.18)
*Moderator variable*									
Coworker Empathic Concern (CEC)	0.14*	0.06	(0.01, 0.24)	0.19**	0.07	(0.03, 0.32)	0.17*	0.07	(0.02, 0.32)
*Interactive effects*									
SOP X CEC	−0.14**	0.04	(−0.22, −0.04)						
OOP X CEC				−0.02	0.05	(−0.10, 0.06)			
SPP X CEC							−0.03	0.06	(−0.14, 0.08)
**Dependent variable model with incivility toward coworker as dependent variable**									
Subordinate gender	−0.14*	0.07	(−0.27, −0.02)	−0.16*	0.07	(−0.30, −0.02)	−0.17*	0.07	(−0.31, −0.03)
Self-oriented perfectionism (SOP)	0.31***	0.06	(0.19, 0.43)	0.17**	0.05	(0.06, 0.27)	0.13*	0.05	(0.02, 0.23)
Ego depletion	0.54***	0.07	(0.40, 0.67)	0.63***	0.07	(0.49, 0.75)	0.65***	0.06	(0.52, 0.77)

N = 253; ***p < 0.001, **p < 0.01, *p < 0.05; CI (95% Confidence Interval for Bootstrapping with 10,000 subsamples).

The result for hypothesis H4 (a) expounded in Table 5. The indirect effect of self-oriented perfectionism on incivility toward coworkers *via* ego depletion was significant when coworker empathic concern was low [indirect effect = 0.25, 95%CI (0.17, 0.35)] as compared to when it was high [indirect effect = 0.09, 95%CI (−0.01, 0.20)], and the indirect effect difference between these two different level settings was significant [indirect effect = −0.16, 95%CI (−0.27, −0.05)], supporting the Hypothesis H4 (a). However, the indirect effect difference of other-oriented perfectionism [indirect effect = −0.03, 95%CI (−0.15, 0.09)] and socially prescribed perfectionism [indirect effect = −0.05, 95%CI (−0.21, 0.12)] on incivility toward coworkers through ego depletion were not significant. Thus, hypotheses H4 (b) and H4 (c) were not supported.

**TABLE 5:** Bootstrapping results for moderated mediation effects.

Variable	Coeff.	SE	95%LLCI	95%ULCI
**Conditional indirect effects as a function of coworker empathic concern**				
*Self-oriented perfectionism*				
Indirect low (−1 SD)	0.25***	0.05	0.16	0.34
Indirect high (1 SD)	0.09	0.05	−0.01	0.20
Difference in indirect effect	−0.16**	0.05	−0.27	−0.05
*Other-oriented perfectionism*				
Indirect low (−1 SD)	0.11*	0.05	0.00	0.19
Indirect high (1 SD)	0.08	0.05	−0.01	0.17
Difference in indirect effect	−0.03	0.06	−0.15	0.09
*Socially prescribed perfectionism*				
Indirect low (−1 SD)	0.06	0.06	−0.05	0.16
Indirect high (1 SD)	0.01	0.06	−0.10	0.13
Difference in indirect effect	−0.05	0.08	−0.21	0.12

N = 253; ***p < 0.001, **p < 0.01, *p < 0.05; LLCI, lower limit confidence interval; ULCI, upper limit confidence interval; CI (95% Confidence Interval for Bootstrapping with 10,000 subsamples).

## Discussion

Based on ego depletion theory, our study examines the mediating mechanism and the boundary conditions of the influences of different dimensions of perfectionism personality on incivility toward coworkers. Our research findings show that ego depletion mediates the relationship between self-oriented perfectionism, other-oriented perfectionism, and incivility toward coworkers. Opposed to our expectation, there is no significant association between socially prescribed perfectionism and incivility toward coworkers *via* ego depletion. It may be because socially prescribed perfectionism is a kind of social standard oriented perfectionism. It more likely causes negative social interaction mechanisms such as stimulating envy and social ostracism from colleagues (Duffy et al., 2012; Riva and Eck, 2016) rather than emotional exhaustion that leads to uncivil behaviors. Another possible reason is that it shows more hostile behaviors in social interactions (Vicent et al., 2017) rather than self-depletion.

In addition, our study also finds that coworker empathic concern only alleviates the mediating effects of ego depletion between self-oriented perfectionism personality and incivility toward coworkers. The moderating effects of coworker empathic concern on the associations between other-oriented perfectionism, socially prescribed perfectionism, and incivility toward coworkers were not significant.

One possible explanation is that the other-oriented perfectionists have high expectations of other people and engage in antisocial behaviors (Stoeber, 2014; Stoeber et al., 2020). These perfectionists would take the care of coworkers of them for granted and even feel that the care is not enough. Thus, empathic concern of coworkers cannot alleviate ego depletion for other-oriented perfectionism employees. Subsequently, the socially prescribed perfectionist is mentally stressed to display perfection and socially disengaged in interpersonal connections (Stoeber et al., 2017), so the essential care and optimistic attitude from coworkers may not effectively contribute to mitigate the negative emotional state of being depleted among such individuals.

### Theoretical Contributions

Our study has three vital theoretical contributions in the existing literature of psychology. First, our study substantially contributes that perfectionism personality serves as a predictor of incivility by considering the employee-coworker interaction perspective in collectivistic Pakistan. In accordance with previous studies (Babalola et al., 2020; Lata and Chaudhary, 2020), personality of individuals profoundly focused on self-interest cognitions by concerning their own psychological needs without caring that the after effects trigger unethical behaviors. Notably, following this research stream, our study finds that all three perfectionism dimensions have positive effects on incivility. Previous studies have considered perfectionism personality as an unidimensional construct and mainly linked it with various detrimental outcomes such as negative attitude (Dang et al., 2020), and abusive behaviors (Guo et al., 2020). Ocampo et al. (2020) called for a more comprehensive understanding of perfectionism personality by examining its dimensional effects on relevant workplace outcomes. Our findings thus enriched the understanding that the dimensions of perfectionism personality that prompt negative impulses, such as incivility toward coworkers, could adversely impact the interpersonal connections. Therefore, our research extended the applicability of individual differentiation characteristics based on perfectionism conceptions to determine behavioral intentions.

Second, another contribution of our study is to explore the mediating role of ego depletion as an emotional reaction path between dimensions of perfectionism personality and incivility of employees toward coworkers. Previous studies on personality traits confirm its validations that neuroticism has strongly predicted incivility at workplace (Milam et al., 2009). In line with the perfectionism studies, existing literature has markedly emphasized that perfectionist individuals are more likely to experience depression through internalized shame (Dorevitch et al., 2020), neglecting that perfectionism personality traits through a resource loss perspective. Therefore, our findings accentuated that employees with perfectionism personality traits are more likely to experience emotional states such as ego depletion. It may further restrict the depleted individuals from following their own intellect, thereby exhibiting inappropriate behaviors such as incivility toward coworkers.

Third, the current study broadens our understanding relevant to the boundary conditions of the impact of perfectionism personality on ego depletion and incivility. Building upon the trait activation theory, we indulged coworker empathic concern as a moderator that may debilitate or strengthen the effects of perfectionism personality (Leonard and Harvey, 2008; Reis and Prestele, 2020). In accordance with the prior research on perfectionism (van der Kaap-Deeder et al., 2016; Guo et al., 2020), our results demonstrated that coworker empathic concern undermines the positive effects of perfectionism personality on ego depletion. In fact, coworkers with higher level of coworker empathic concern are more conscious and express extra care toward depleted individuals, who exhibit self-oriented perfectionism and thereby lessens incivility behaviors. Besides, our findings also revealed that coworker empathic concern does not moderate the relationship between other-oriented, socially prescribed perfectionism and ego depletion. Therefore, the present research extended the existing knowledge by emphasizing the positive attitude from coworkers to reduce the psychological state of an individual of being depleted in relation to perfectionism personality and incivility toward coworkers.

### Practical Implications

Our study massively contributes by providing various practical implications for organizational practitioners to take necessary measures to obstruct incivility behaviors. First, our study depicts that employee perfectionism personality plays a critical role in shaping incivility of employees toward coworkers. Given that perfectionistic individuals are more inclined to experience fear, anxiety, and depression (Leonard and Harvey, 2008), occupational health researchers should concentrate on the shortcomings of the perfectionism personality that harms the well-being of employees and hampers organization growth. It is obligatory for the organization practitioners to create a learning climate in which employees are trained to set rational goals and to strive for them and are capable enough to develop strong interactions with coworkers, which ultimately reduce the negative reactions like incivility.

Second, the depleted individuals are unlikely to convey rude behaviors against the ethical norms of the organization (Yam et al., 2014), yet are lacking of self-regulatory resources instigated by his or her own perfectionism personality. Therefore, the employees are motivated to participate in employee well-being programs arranged by the organization, such as programs concerning employee safety and health, stress management, and physical activities to boost the energy level. Although all of these productive activities can benefit the individuals in exercising their mental abilities properly, they lessen their negative emotional reactions such as feeling depleted and reducing negative behaviors such as incivility.

Third, organizations should encourage employees to exhibit high empathic concern, carefully design their course of actions, and display a generous and polite attitude during interpersonal interactions. Our results illustrate that coworker empathic concern influences the ego depletion of the employee, given that trait activator is fruitful to alleviate the negative feelings caused by perfectionism personality which relates positively with incivility toward coworkers.

### Limitations and Directions for Future Research

Our study has several limitations. Firstly, we only investigated ego depletion as a mediator between perfectionism personality and incivility toward coworkers. Future studies may integrate subjective fatigue (Yam et al., 2014), and sleep disturbance (Akram et al., 2020) as mediating factors to assess the impact of perfectionism personality and relevant outcomes such as incivility. For example, Yam et al. (2014) had incorporated dual process theory to investigate the mediating mechanism of subjective fatigue in relationship between ego depletion and unethical behaviors, while Akram et al. (2020) had merged the sleep related cognitions as a mediating variable in association of multidimensional perfectionism and negative outcomes such as insomnia symptoms.

Secondly, our research constitutes empathic concern as the moderator. Future studies may incorporate other constructs such as perspective taking (Batanova and Loukas, 2011). Thirdly, data was mainly collected from the telecommunication sector in Pakistan. Subsequently, the sample from different sectors such as textile, education, and transport would enrich the understanding of perfectionism and incivility studies at different levels. The generalizability of our research may be validated by deploying a comparative study approach to explore the cross-cultural differences in different settings, i.e., Asian or Western, by extending the existing knowledge on perfectionism and incivility studies.

## Conclusion

This study primarily centered on ego depletion theory to clarify the relationships between perfectionism personality dimensions and incivility toward coworkers and integrated the mediating mechanism of ego depletion. Remarkably, the study provides a considerate viewpoint that perfectionism personality could strongly predict the individual behavioral consequences and attenuates the strength of interpersonal connections. Further, we accentuated how coworker empathic concern plays a prominent role in hostile situations to mitigate the negative feelings of the employee of being depleted caused by the perfectionism personality. Our study extended the literature by covering existing research gaps by integrating the moderated mediation model with ego depletion and trait activation theory to comprehend incivility behaviors as a new viewpoint.

## Data Availability Statement

The raw data supporting the conclusions of this article will be made available by the authors, without undue reservation.

## Ethics Statement

The studies involving human participants were reviewed and approved by approval was granted by the Dean of School of Management and Economics, University of Electronic Science and Technology, China; No. 20200120. The patients/participants provided their written informed consent to participate in this study.

## Author Contributions

MAH contributed in writing the original draft, data collection, and design of the study. LC contributed in conceptualization, developed the theory, and edited the manuscript. LW researched the statistical methods, performed computations, and verified the analytical methods. All authors contributed to the article and approved the submitted version.

## Conflict of Interest

The authors declare that the research was conducted in the absence of any commercial or financial relationships that could be construed as a potential conflict of interest.

## Publisher’s Note

All claims expressed in this article are solely those of the authors and do not necessarily represent those of their affiliated organizations, or those of the publisher, the editors and the reviewers. Any product that may be evaluated in this article, or claim that may be made by its manufacturer, is not guaranteed or endorsed by the publisher.
